# Individual and Combined Effects of Fumonisin B_1_, Deoxynivalenol and Zearalenone on the Hepatic and Renal Membrane Lipid Integrity of Rats

**DOI:** 10.3390/toxins10010004

**Published:** 2017-12-22

**Authors:** András Szabó, Judit Szabó-Fodor, Hedvig Fébel, Miklós Mézes, Krisztián Balogh, György Bázár, Dániel Kocsó, Omeralfaroug Ali, Melinda Kovács

**Affiliations:** 1 Institute of Diagnostic Imaging and Radiation Oncology, Kaposvár University, 7400 Kaposvár, Hungary; 2 “MTA-KE Mycotoxins in the Food Chain” Research Group, Hungarian Academy of Sciences, Kaposvár University, 7400 Kaposvár, Hungary; fodor.judit@ke.hu (J.S.-F.); kocso.daniel@ke.hu (D.K.); kovacs.melinda@ke.hu (M.K.); 3 Research Institute for Animal Breeding, Nutrition and Meat Science, National Agricultural Research Center, 2053 Herceghalom, Hungary; febel.hedvig@atk.naik.hu; 4 Department of Nutrition, Faculty of Agricultural and Environmental Sciences, Szent István University, 2013 Gödöllő, Hungary; mezes.miklos@mkk.szie.hu (M.M.); balogh.krisztian@mkk.szie.hu (K.B.); 5 Faculty of Agricultural and Environmental Sciences, Kaposvár University, 7400 Kaposvár, Hungary; bazar@agrilab.hu (G.B.); omeralfaroug.ali@gmail.com (O.A.)

**Keywords:** rat, liver, kidney, fusariotoxins, multitoxic effects, phospholipids

## Abstract

(1) Background and (2) Methods: A 14-day in vivo, multitoxic (pure mycotoxins) rat experiment was conducted with zearalenone (ZEA; 15 μg/animal/day), deoxynivalenol (DON; 30 μg/animal/day) and fumonisin B_1_ (FB_1_; 150 μg/animal/day), as individual mycotoxins, binary (FD, FZ and DZ) and ternary combinations (FDZ), via gavage in 1 mL water boluses. (3) Results: Body weight was unaffected, while liver (ZEA↑ vs. DON) and kidney weight (ZEA↑ vs. FDZ) increased. Hepatocellular membrane lipid fatty acids (FAs) referred to ceramide synthesis disturbance (C20:0, C22:0), and decreased unsaturation (C22:5 n3 and unsat. index), mainly induced by DON and to a lesser extent by ZEA. The DON-FB_1_ interaction was additive on C20:0 in liver lipids. In renal phospholipids, ZEA had the strongest effect on the FA profile, affecting the saturated (C18:0) and many n6 FAs; ZEA was in an antagonistic relationship with FB_1_ (C18:0) or DON (C18:2 n6, C20:1 n9). Hepatic oxidative stress was the most expressed in FD (reduced glutathione and glutathione peroxidase), while the nephrotoxic effect was further supported by lipid peroxidation (malondialdehyde) in the DON treatment. (4) Conclusions: In vivo study results refer to multiple mycotoxin interactions on membrane FAs, antioxidants and lipid peroxidation compounds, needing further testing.

## 1. Introduction

The contamination of cereals with mycotoxins is a worldwide problem. Although the diversity of mycotoxins is enormous, a rather important sub-group is the Fusarium toxins, produced by divergent fungal species of the *Fusarium* genus. Fusarium mycotoxins are the most frequently occurring mycotoxins worldwide and more specifically fumonisin B_1_ (FB_1_), deoxynivalenol (DON) and zearalenone (ZEA) are the most likely to co-occur [1,2,3,4,5]. 

For the different individual fusariotoxins, the target organs, the mode of toxic action and the physiological indicators have been established in detail. Very briefly, FB_1_ has a long-chain hydrocarbon unit, and disturbs or inhibits ceramide synthesis with further, cell membrane homeostasis associated consequences in vertebrates. Besides ceramide, rat hepatic lipids (phospholipid classes and their fatty acids) were shown to be affected in vivo [6]. DON causes a broad variety of toxic effects in animals; the primary effect at the cellular level is the inhibition of protein synthesis by binding to the ribosomal subunit, DON being also immune-suppressive and inducing apoptosis [7]. ZEA competes with the natural estradiol-17β for binding sites (estradiol receptors) in both males and females, and obstructs normal steroid hormone (estradiol, testosterone, and progesterone) synthesis, acting as an endocrine disruptor [8]. 

Fusariotoxins are diverse and it has been reported that their co-occurrence is a vital problem, namely corn has been shown to be co-contaminated with FB_1_, DON and ZEA [9]. The combined, multitoxic effect of these three mycotoxins was first tested on yeast, describing low-dose associated DON↔ZEA antagonism, providing a dose-dependent shift to synergism [10]. In a nematode (*C. elegans*), FB_1_, DON and ZEA compromise survival, lifespan, growth and reproduction; in the latter case, FB_1_ and DON acted as synergists [10]. In an in vitro test on BRL 3A rat liver cells, cell viability was assayed with MTT test [11]. The cytotoxic effect was the following: DON > aflatoxin B_1_ (AFB_1_) > ZEA > FB_1_, exerted via emerging reactive oxygen (ROS) species production and apoptosis; synergism was proven for the mixtures AFB_1_ + ZEA and AFB_1_ + DON. In porcine kidney cell line 15 (PK-15), nephrotoxicity was assayed, and the individual toxicity order was the following: DON > AFB_1_ > ZEA > FB_1_. Synergism was found for DON and ZEA + AFB_1_ concerning cytotoxicity; however, in ternary combination, AFB_1_ and ZEA were antagonists. Moreover, in an in vitro test, HepG2 cells were exposed to FB_1_, DON and ZEA; results indicate that ZEA and DON induce severe apoptosis, as compared to FB_1_, but possible interactions were not reported [12]. These fusariotoxins were further tested in porcine granulosa cells [13], swine jejunal epithelial cells [14] and porcine whole-blood cells [15], with mostly concordant results in terms of cytotoxicity, ROS production, apoptosis (and ultimately necrosis) induction in the following order: DON > ZEA > FB_1_. 

Newest results applying RT-PCR provide evidence that the above fusariotoxins as single-agents (individual toxins) possess large interactomes and those clearly describe the associated predicted secondary signaling responses [12].

In the aforementioned surveys, the mycotoxin concentrations were usually low. Acute toxicity is rarely occurring, thus chronic exposure to low concentrations can be of high importance for human and animal health [16]. In this study, we tested the in vivo effects of single and combined purified fusariotoxins, seeking for possible individual toxic effects and interactions in the target organs, i.e., liver and kidney.

## 2. Results

### 2.1. Bodyweight and Daily Feed Intake

The initial and final bodyweight (BW), as well as the daily and the total cumulative feed intake were not different among the eight groups, as analyzed with ANOVA and “post hoc” test (Table 1). Consequently, BW gains, either total (Σ for 14 days) or the daily values, were also not different among the eight groups. No mortality or abnormal symptoms were observed during the trial.

### 2.2. Organ Weights (Absolute and Relative)

As shown in Table 1, there was a significant difference in the absolute liver weight between the DON and the ZEA groups (DON providing the lower values), but, for all other groups, means were not different from these two or each other. The relative liver weight (percent of body mass) was significantly lower in the DON treated group, as compared to the FB_1_ and ZEA as single mycotoxin treatments, and FD and DZ as binary treatment mean values, while the other groups were not different from any of these, as tested with ANOVA.

The interaction of the different mycotoxin treatments was evaluated with the Bliss independence method. According to the calculations, there was an antagonistic effect between DON and ZEA on the absolute liver weight. Analyzing DON, its effect on the relative liver weight was antagonistic with that of FB_1_ and ZEA, at significant levels in both cases.

The absolute kidney weight was the lowest in the FDZ group, but this was only significantly different from the mean of the ZEA group (Table 1). In the relative kidney weight, no inter-group differences were detected, thus, for the mentioned traits, toxin interactions were not considered. The spleen provided a significant difference in its relative weight between DON, and FZ and DZ groups, the latter two providing higher values. 

### 2.3. Hepatic Phospholipid Fatty Acid Composition

As analyzed with one-way ANOVA, three individual fatty acids (and unsaturation index, UI) provided inter-group differences (Table 2) in the hepatic phospholipid (PL) fatty acid (FA) profile. Arachidic acid (C20:0) proportion was lower in the FB_1_, DON and FD groups, as compared to the control. Behenic acid (C22:0) proportion was higher in the control group, as compared to the DON and ZEA group mean values, while docosapentaenoic acid (C22:5 n3) proportion was lower in the DON group, as compared to FD. From calculated FA indices, the unsaturation index (UI, the number of double bonds in 100 acyl chains) was significantly lower in the FB_1_ and ZEA groups, as compared to the DZ treatment.

When the DON-FB_1_ interaction was analyzed on the proportion of arachidic acid, an additive effect was found. FB_1_ and ZEA were in synergistic relationship in lowering the UI in the hepatic PL FA profile.

**Figure 1:**
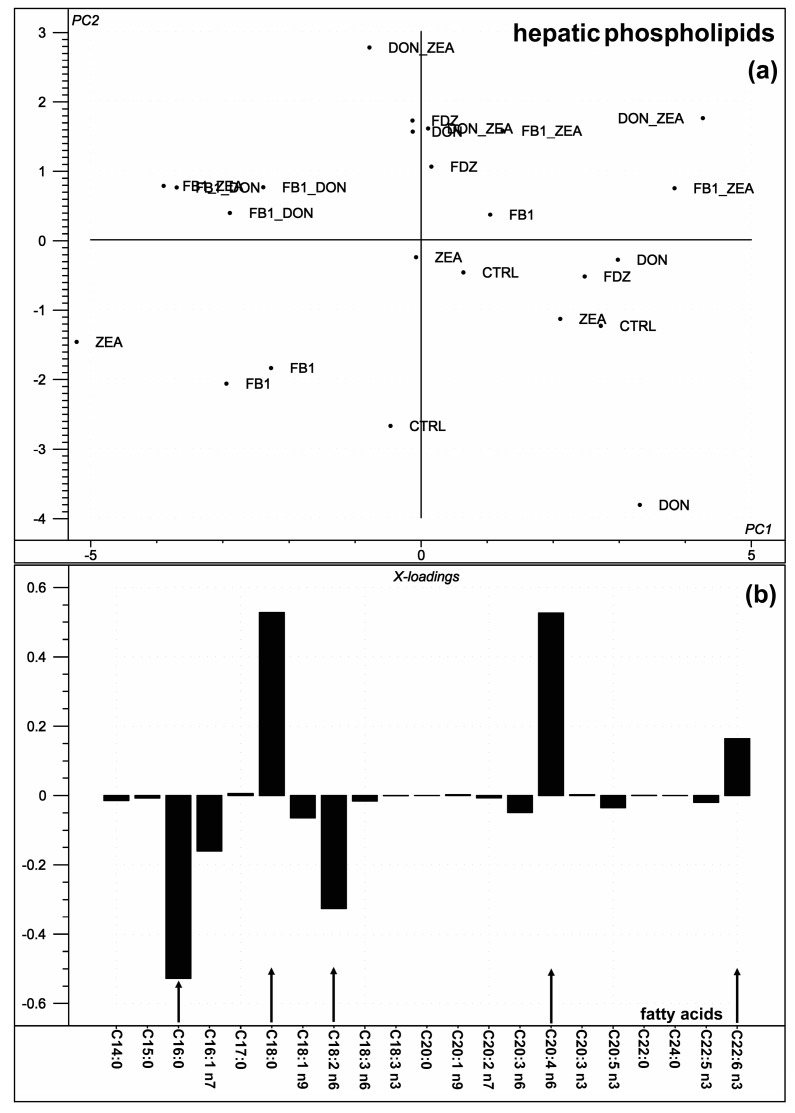
Results of the principal component analysis performed on the compositional data of the liver PL fatty acids. (**a**) Score plot shows the orientation of the samples belonging to the different toxin treatments (CTRL: control) in the plane of the 1st and 2nd principal components (PC1 and PC2, respectively), where PC1 and PC2 are influenced by the multivariate data of the liver PL fatty acids. PC1 and PC2 explain 58% and 31% of the total variance of the liver PL fatty acids, respectively. (**b**) Loading bar graph of the PC1 shows the contribution of the individual liver PL fatty acids to the newly developed latent variable: the higher the loading value, the higher impact of the variance of the respective fatty acid on the variance of PC1.

When performing principal component analysis (PCA) on the individual fatty acids (calculated FA variables excluded in the first attempt), there were five (palmitic acid, C16:0; stearic acid, C18:0; linoleic acid, C18:2 n6; arachidonic acid, C20:4 n6 and docosahexaenoic acid, C22:6 n3) FAs with a marked contribution to the variation of the original data (Figure 1a,b), as described by PC1 along the abscissa. Analyzing the calculated FA indices with PCA, UI was the only component providing a strong contribution (95% of the total) to the variance (not plotted). There was no clear separation of the different mycotoxin treatments found within the PCA score plot (Figure 1a), but the PC1 loading (Figure 1b) shows that C16:0, C18:0, C18:2 n6, C20:4 n6, and C22:6 n3 contributed the most to the total variance of the investigated fatty acids of liver.

### 2.4. Kidney Phospholipid Fatty Acid Composition

In the renal total phospholipid FA profile, five individual FAs provided detectable inter-group differences with ANOVA, while no calculated variables were different among groups (Table 3). As compared to the control, the FD treatment significantly increased the proportion of myristic acid (C14:0). For stearic acid (C18:0), FB_1_ provided an increasing effect, as compared to ZEA. Linoleic acid (C18:2 n6) was significantly increased by ZEA, as compared to the DZ treatment, while gamma-linoleic acid (C18:3 n6) was also increased by ZEA, as compared to the control. ZEA further increased the proportion of gondoic acid (C20:1 n9), as compared to the DON and the FD group means.

When the Bliss independence method was applied to elucidate interactions, ZEA and FB_1_ were found to act antagonistically on the stearic acid proportion. In the case of gondoic acid, both DON and ZEA were found to act as antagonists, as well as FB_1_ and ZEA (FB_1_ as a co-occurring factor in the FD treatment).

**Figure 2:**
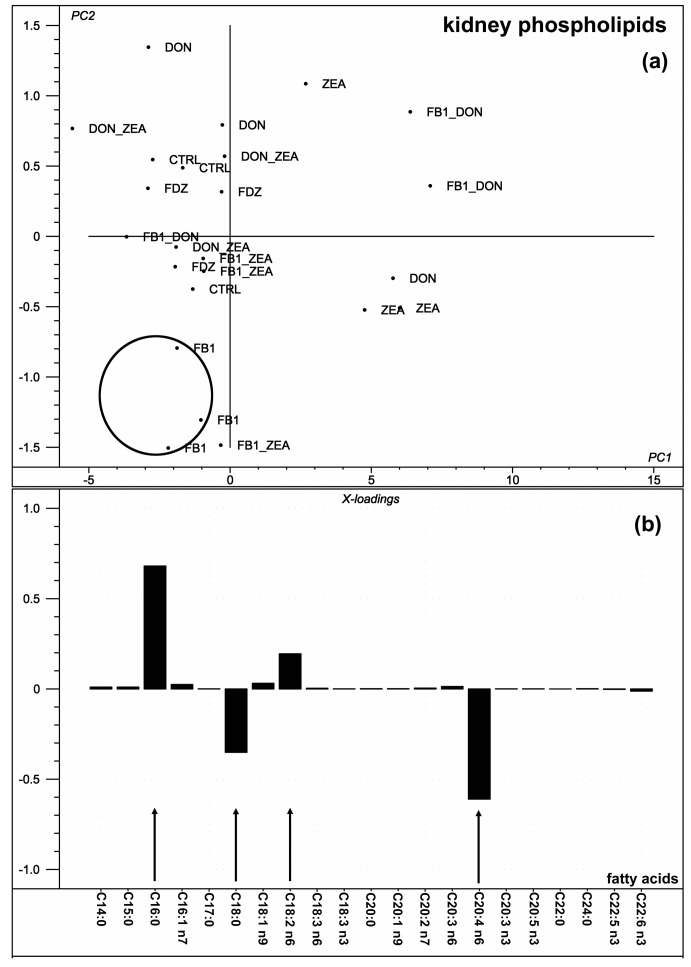
Results of the principal component analysis performed on the compositional data of the kidney PL fatty acids. (**a**) Score plot shows the orientation of the samples belonging to the different toxin treatments (CTRL: control) in the plane of the 1st and 2nd principal component (PC1 and PC2, respectively), where PC1 and PC2 is influenced by the multivariate data of the kidney PL fatty acids. PC1 and PC2 explains 86% and 6% of the total variance of the kidney PL fatty acids, respectively (circle is only a visualization aid). (**b**) Loading bar graph of the PC1 shows the contribution of the individual kidney PL fatty acids to the newly developed latent variable: the higher the loading value, the higher impact of the variance of the respective fatty acid on the variance of PC1.

In the renal PL FA profile, the PC analysis provided a result in which PC1 explained 91.5% of the variation of the individual FA data (Figure 2a). 

Only four FAs played a determinant role in this, namely palmitic (C16:0), stearic, linoleic and arachidonic acids (Figure 2b). Analyzing the FA-based indices (separated from the individual FA results), only the UI played a determinant role in determining the variation described by PC1, explaining 95% of the total, similar to the liver. Similar to Figure 1, there was no clear separation of the different mycotoxin treatments found within the PCA score plot (Figure 2a), although the three replicates of FB_1_ treatment clustered—as indicated with a circle. The PC1 loading (Figure 2b) shows that C16:0, C18:0, C18:2 n6, and C20:4 n6 contributed the most to the total variance of the investigated fatty acids of kidney.

### 2.5. Liver Antioxidant and Oxidative Parameters

In the liver samples, as compared to the control, from the single toxin treatment ZEA, all binary combinations (FD, FZ and DZ) significantly increased the reduced glutathione (GSH) concentration (Table 4). The ternary combination (FDZ) had an identical effect. In contrast, glutathione peroxidase (GSHPx) activity was increased only by the FD treatment, as compared to the control with ANOVA. 

The binary interactions (Bliss independence method) were found to be antagonistic on the level of GSH in the following cases: FB_1_↔DON, FB_1_↔ZEA and DON↔ZEA. FB_1_ and DON exerted antagonistic effects on the activity of GSHPx.

Meanwhile, malondialdehyde (MDA) concentration was not different among groups, when analyzing the antioxidant and lipid peroxidation parameter results with PCA, the only compound providing marked effect on the variance of PC1 was MDA (explained variance: 99.3%; results not plotted).

### 2.6. Kidney Antioxidants and Oxidative Parameters

In the kidney samples, malondialdehyde (MDA) concentration was elevated by the DON treatment, as compared to DZ and FZ. Neither GSH nor GSHPx showed any alteration in the kidney (Table 4). The interaction between DON and ZEA was antagonism on the MDA concentration, while between FB_1_ and DON it was synergistic, without significance.

## 3. Discussion

### 3.1. Bodyweight and Daily Feed Intake

The individual fusariotoxin treatments and their combinations failed to induce any alteration in the feed intake and gross somatic parameters. The 14-day study can be characterized as a mid-term treatment. DON at the applied dose had no detectable effect on feed intake or BW change, while Rotter et al. [17] published that the main effect of DON at dietary concentrations of 12–20 mg/kg feed (ca. 15-fold higher than the present treatment) is the reduction in feed consumption in rats. The applied DON dose (30 µg/kg BW) was equivalent to the mean value of the chronic exposure level defined by the EFSA [18], ranging between 3.9 and 43.3 µg/kg BW. Since the other mycotoxins or combinations exerted no effect on these traits, their detailed discussion is void, while it is interesting to note that subchronic FB_1_ exposure may compromise growth in rats (at 10-fold the present dose [19]).

### 3.2. Organ Weights (Absolute and Relative)

The difference in the absolute liver weight between the DON and the ZEA groups (DON < ZEA) is a finding that may be explained by the hepatotoxic effect of ZEA in rodents at low mycotoxin concentration (10 µg/kg BW for 14 days) [20]. Similar background may exist for the relative liver weight, adding that relative weight change has a mere hepatic background, since final BW was identical in all groups. Testing lactate dehydrogenase leakage in hepatic cell lines, Wentzel et al. [12] found that ZEA and even FB_1_ exert similar, but slight damaging effects. However, the multitoxic arrangements revealed a proven antagonistic effect between DON and ZEA on the absolute liver weight, and also antagonism between DON and FB_1_, ZEA, FD and DZ. This suggests that the two mycotoxins (DON vs. ZEA) exert their toxic action via different interactome sections. Wentzel et al. [12] reported in an in vitro–in silico approach that the expression of tumor suppressor protein 53 gene is down-regulated by FB_1_, as compared to DON and ZEA. This refers to a moderate level of apoptosis and compromised cell metabolism (including ageing) in the FB_1_ group. This is also supported by the findings of Gelderblom et al. [6], when a 21-day FB_1_ feeding had a significant effect on rat liver weight only by a 50-fold higher exposure level, as compared to this study. Wentzel et al. [12] stipulated that in vitro the malignant colonization of hepatocytes is occurring through different pathways in a toxin-type dependent manner. Moreover, from the DON-FB_1_-ZEA triplet ZEA was the only mycotoxin that up-regulated the DNA and RNA cytosine deaminase enzyme family and the activation-induced cytidine-deaminase enzyme in Caco-2 cells, referring to associations to cell-to-cell signaling, cell growth and proliferation.

When analyzing the kidney weight (ZEA < FDZ), it can again be hypothesized that ZEA acts differently from DON (A type trichothecene), probably due to the exclusive renal excretion of multiple, hydroxylated metabolites of DON [8]. A similar phenomenon was found for the relative spleen weight: the DON–ZEA interaction on the relative spleen weight was synergistic, with a DON < FZ and DZ difference among the group means. The background of the latter results found in relationship with the action of ZEA may be associated as well with the fact that ZEA [21] and even DON [17] are immunotoxic.

### 3.3. Hepatic Phospholipid Fatty Acid Composition

Arachidic acid (C20:0) proportion was lower in the FB_1_, DON and FD groups, as compared to the control, with a significant DON + FB_1_ additive effect because FB_1_ and DON attack a quasi-inert fatty acid embedded into the cellular membrane structure that might only be viable if arachidic acid plays some crucial role (being precursor along the FA metabolism) or strictly co-occurs with a biologically (more) active component. The former may be true, since arachidic acid itself is not a major membrane lipid component, but is the precursor of behenic and lignoceric acids (C22:0 and C24:0). In the present work, behenic acid (C22:0) proportion was higher in the control group, as compared to the ZEA treatment. Saturated fatty acids are not sensitive towards lipid peroxidation, but are vital components of ceramides. As earlier reported in a study on rabbits, red cell membrane was markedly depleted in behenic (C22:0) acid by FB_1_ [22]. This lowering effect has been explained by the hypothesis that behenic acid is an important component of sphingomyelins, located mostly in the outer leaflet of the membrane bilayer. This fraction is hydrolyzed by the neutral Mg^2+^-dependent sphingomyelinase enzyme to phosphorylcholine and ceramide [23]. Thus, decreasing behenic acid proportion refers to ceramide synthesis inhibition, the characteristic mode of action of FB_1_ mycotoxin, as also attained partly in the FB_1_ and DON synergism.

Among the polyunsaturated omega-3 FAs, docosapentaenoic acid (C22:5 n3, DPA) was decreased in the DON group, as compared to the FD, while FB_1_ and DON were found to act as antagonists in this case. There is no specific toxicological explanation for the DON-induced lowering of the DPA proportion, or fumonisin B_1_, as a DPA increasing factor, but DON has been published to be strongly related to interleukin-6 (IL-6) expression in nephropathy, which has been markedly suppressed by DPA [24]. Interestingly, the IL-6 expression inhibition has been extended later to peritoneal macrophages and further omega-3 FAs, including DHA ([25], as also supported by our PCA loading plot results in Figure 1).

### 3.4. Kidney Phospholipid Fatty Acid Composition

In the renal total phospholipids, FD treatment significantly increased the myristic acid (C14:0) proportion. Since FB_1_-DON interaction was not found, this result may be a simple toxic effect referring to a slight perturbation of the saturated FAs, synthesized de novo. Although palmitic acid was not responsive, stearic acid (C18:0) proportion increased as an effect of FB_1_, despite the (here appearing) proven FB_1_↔ZEA interaction (antagonism). The stearate accretion in multiple cellular compartments (which were not separated in our approach) is known: Venkataraman et al. [26] reported in human kidney cells that, as a result of the upstream of growth and differentiation factor 1, the cells continue ceramide synthesis even in the presence of FB_1_, but ceramide is in this case channeled into neutral glycosphingolipids. Authors also confirmed the stearic acid enrichment, which was even further increased by FB_1_. It is thus highly probable that FB_1_ induced a saturated FA synthesis, with minor interactions, mostly as a single agent.

From within the n6 FAs linoleic acid (C18:2 n6) proportion was significantly increased by ZEA, as compared to the DZ treatment, while gamma-linoleic acid (C18:3 n6) was as well increased by ZEA, as compared to the control. However, the linoleic acid proportion was only different between the ZEA and the DZ treatment, but DON-ZEA antagonism was not proven. Since linoleic acid is essential, its membrane lipid accretion might be a consequence of its increased dietary uptake, but feed intake was not altered by any of the treatments. Thus, taking as well the gamma-linoleic acid increase into consideration, a slight shift of n6 fatty acid metabolism was hypothesized, which did not influence arachidonic acid proportion. The only supportive data published on the effects of fusariotoxins and linoleic acid refer to the fact that in rat kidney linoleic acid administered with a trichothecene type toxin (Roridin E) augments toxicity via an increased membrane incorporation of both compounds [27]. However, it was a novel finding that linoleic acid, probably due to its high membrane proportion, played a determinant role in shaping the variance determined by PC1 (Figure 2b).

### 3.5. Liver Antioxidant and Oxidative Parameters

The tissue concentration of the main, low molecular weight antioxidant, GSH has been increased by ZEA and all binary and the ternary combinations. However, glutathione peroxidase (GSHPx) activity was only different between control and FD, but FB_1_ and DON were found to act antagonistically. Based on the above facts, ZEA is the main of the applied three toxins in influencing the glutathione redox system, evoking even the enzymatic defense. This has been first indicated in vitro in Hep G2 cells [28], describing ZEA as the root compound of oxidative stress. Hassen et al. [28] found that oxidative damage is likely to be evoked as one of the main pathways of ZEA toxicity and may be an initiation to the mechanism of ZEA in its different genotoxic and cytotoxic effects. Our results confirm this finding, since the ultimate product of lipid peroxidation, malondialdehyde was not different among groups; ultimately, ZEA played a hepatotoxic role, of which the extent was compensated by the increase of the non-enzymatic antioxidant (GSH), up to a severity that it already activated antioxidant enzyme response, but remained without cytotoxic end-product (MDA) concentration elevation.

### 3.6. Kidney Antioxidants and oxidative Parameters

The renal oxidation parameters were different from those in the liver, in that DON increased the MDA concentration, as compared to the DZ and FZ; the interaction on MDA between DON and ZEA was antagonistic. To the authors best knowledge this is the first in vivo multitoxin exposure study in which DON has been found to exert defined lipid peroxidation in the renal cortex. The study of Liang et al. [29] revealed that the combination of DON and ZEA exhibited sub-additive, while Kouadio et al. [30] showed more than additive nephrotoxic effect of DON and FB_1_ in mice, probably in a species-associated manner. Moreover, in the cited study, the dose of DON was higher and that of FB_1_ was lower (45 and 110 µg/kg BW/day, respectively) than in the present one and a shorter exposure period (seven days) was applied. Oxidative stress occurs when oxygen free radical formation exceeds the antioxidant capacity, thus it is rather uncommon that non-enzymatic (GSH) and enzymatic (GSHPx) defense has been not found to be activated, while lipid peroxidation end-product (MDA) concentration increased. It is likely that the enzymatic adaptation of splanchnic organs needs a longer exposure interval.

## 4. Conclusions

In a novel in vivo, multitoxic approach, rats were exposed to ZEA, DON and FB_1_, as individual mycotoxins, as well as in their binary and ternary combinations, for 14 days, via gavaging a single toxin bolus per day. Bodyweight was not affected, while absolute liver (ZEA↑ vs. DON) and kidney weight (ZEA↑ vs. FDZ) was modified. The hepatocellular membrane lipid fatty acid profile referred to ceramide synthesis disturbance (C20:0, C22:0), and showed decreased unsaturation (UI), mainly provoked by DON and to a lesser extent by ZEA. In renal phospholipids, ZEA had the strongest effect on the FA profile, affecting stearic acid and most n6 FAs; ZEA was, in most cases, in an antagonistic relationship with FB_1_ or DON. Summarizing toxicity, hepatic oxidative stress was the most expressed by the FD treatment, while nephrotoxic effect was proven by malondialdehyde in the DON treatment. Liver phospholipids were the most sensitive on DON (and partially on FB_1_ and DON), oxidative stress was mostly induced by FB_1_ and DON, while ZEA was affecting organ weight. Renal lipids were affected primarily by ZEA, providing in numerous cases antagonism with FB_1_, while renal lipid peroxidation was mostly provoked by DON. Principal component analysis proved to be effective in the identification of the FAs contributing with the highest relative power to the overall variance. Results refer to multiple and sometimes diverse interactions of the analyzed fusariotoxins, needing further analysis on in vivo models.

## 5. Materials and Methods

### 5.1. Animals and Feeding

Adult, male Wistar Crl:WI BR rats (8 weeks of age) were enrolled in the study and were kept in metabolic cages (Tecniplast, Castronno, Italy) individually. The animals (*n* = 3/group, total *n* = 24) were fed Ssniff R/M-Z+H feed (Ssniff GmbH, Soest, Germany; Table 5). The rats were kept in a 12-h light and 12-h dark daily rhythm, at 20 °C in a rodent room. The relative air humidity was 50%. Feed was offered ad libitum, and feed intake was logged daily (from the daily feed consumption recordings). 

The mycotoxins were purchased from Sigma-Aldrich (Schnelldorf, Germany), and stock solutions were prepared with double-distilled water. The solutions contained the daily toxin dose in exactly 1 mL, and this solution was administered as a single gavage dose. For the control animals, 1 mL of double distilled water was dosed. Mycotoxin treatment was set as follows for individual toxins: FB_1_: 150 µg/animal/day, DON: 30 µg/animal/day and ZEA: 15 µg/animal/day, as single gavage boluses, every day at 8:00 AM. The applied doses were pre-determined according the EU limits in finished feed for young pigs (in the absence of limits for rat feed; based on the European Commission Recommendation 2006/576/EC [31]. The toxins binary (FD, FZ, DZ) and ternary (FDZ) mixture meant the same mycotoxin exposure (all mycotoxins in a single, 1 mL aliquot) in an additive manner. The entire treatment lasted for 14 days. After this, animals were sacrificed by cervical dislocation and were immediately exsanguinated and dissected. Total dissected fresh livers and kidneys were weighed immediately and samples were stored frozen (−80 °C) in Eppendorf tubes until analysis. Absolute weight was the fresh total organ weight; relative weight means the organ weight as a percentage of the total BW.

The experimental protocol was authorized by the Food Chain Safety and Animal Health Directorate of the Somogy County Agricultural Office, under the permission number SOI/31/1679-11/2014.

### 5.2. Lipid Analysis

Liver and kidney samples of ca. 300 mg were homogenized (IKA T25 Digital Ultra Turrax, Staufen, Germany) in 20-fold volume of chloroform:methanol (2:1 *v:v*) and total lipid content was extracted according to Folch et al. [32]. Solvents were ultrapure-grade (Sigma-Aldrich, Schnelldorf, Germany) and 0.01 % *w:v* butylated hydroxytoluene was added to prevent fatty acid oxidation. For the separation of lipid fractions, extracted total lipids were transferred to glass chromatographic columns, containing 300 mg silica gel (230–400 mesh) for 10 mg of total lipids [33]. Neutral lipids were eluted with 10 mL chloroform for the above fat amount, then 15 mL acetone:methanol (9:1 *v:v*) was added, while 10 mL pure methanol eluted the total phospholipids. This latter fraction was evaporated under a nitrogen stream and was transmethylated with a base-catalyzed NaOCH_3_ method [34]. Fatty acid methyl esters were extracted into 250 μL ultrapure n-hexane for gas chromatography, which was performed on a GCMS-QP2010 SE apparatus (AOC 20i automatic injector), equipped with a Phenomenex Zebron ZB-WAX Capillary GC column (30 m × 0.25 mm ID, 0.25 micrometer film, Phenomenex Inc., Torrance, CA, USA). Characteristic separation conditions were: injector temperature: 270 °C, detector temperature: 300 °C, helium flow: 28 cm/s. The oven temperature was graded: from 80 to 205 °C: 2.5 °C/min; 5 min at 205 °C; from 205 to 250 °C 10 °C/min; and 5 min at 210 °C. The makeup gas was nitrogen. To identify individual FA, an authentic external FA standard (37 Component FAME Mix, Sigma-Aldrich, Cat. No.: CRM47885) was used. Fatty acid results were expressed as weight percent of total fatty acid methyl esters.

### 5.3. Analysis of Lipid Peroxidation

Lipid peroxidation (end-phase) was assessed by the determination of malondialdehyde (MDA) levels using the 2-thiobarbituric acid method from raw tissue samples [35]. The concentration of reduced glutathione (GSH) was measured as non-protein thiols by Ellmann’s reagent [36] and the activity of glutathione peroxidase (GSHPx) was determined according to Lawrence and Burk [37] in the 10,000 g supernatant fraction of 1:9 (*w:v*) tissue homogenate in physiological saline. Protein content of the supernatant was determined with the Lowry method [38], using Folin phenol reagent. All analyses were performed from tissue samples after storage at −80 °C.

### 5.4. Statistical Analysis

For the comparison of group means of somatic traits (bodyweight (BW), organ weights, relative organ weights and feed intake), fatty acid profile, peroxidation products and antioxidant parameters univariate ANOVA was used with Tukey “post hoc” test, with the SPSS 20 software (2012) [39]. In cases where there existed significant difference between two or multiple groups, the Bliss independence method was applied to ascertain possible, mycotoxin-treatment associated interactions [40]. It is based on the principle that drug effects are outcomes of probabilistic processes and assumes that drugs act independently in such a manner that neither of them interferes with the other (different sites/modes of action), but each contributes to a common result. The observed combination effect expressed as a probability (0 ≤ E_AB_ ≤ 1) can be compared to the expected additive effect given by the common formula for probabilistic independence:

E_A_ + E_B_(1 − E_A_) = E_A_ + E_B_ − E_A_E_B_, where 0 ≤ E_A_ ≤ 1 and 0 ≤ E_B_ ≤ 1. (Only the significantly existing interaction results are shown in a textual form, merely for the cases where ANOVA also provided significant inter-group differences.)

Afterwards, Principal Component Analysis (PCA) was performed on the fatty acid profile of the liver and the kidney, as well as on the blood biochemical parameters with the Unscrambler 9.7. software [41], to seek principal components describing the variance being responsible for the “group formation” with the highest possible efficacy. The sole purpose of PCA was not to discriminate the certain groups of treatments based on the chemical composition, but to describe the basic orientation of the groups within the multidimensional space described by the variables investigated (e.g., FA profile), and to detect those variables (chemical components, FAs) that have the largest variance compared with the others. The orientation of the samples is described by the score plot showing the scores of each sample along the first two principal components. The variable impact is presented with the loadings bar graph that shows the contribution of the variance of each investigated variable to the variance of the first principal component, i.e., values of the loadings graph are the weights for each original variable when calculating the principal component.

## Figures and Tables

**Table 1:** Somatic and feed intake data of the experimental animals (FD: FB_1_ + DON; FZ: FB_1_ + ZEA; DZ: DON + ZEA; FDZ: FB_1_ + DON + ZEA).

Group	Control	FB_1_	DON	ZEA	FD	FZ	DZ	FDZ
*Somatic traits*	Mean ± SD	Mean ± SD	Mean ± SD	Mean ± SD	Mean ± SD	Mean ± SD	Mean ± SD	Mean ± SD
BW initial (g)	307.2 ± 14.9	306.4 ± 9.49	299.6 ± 4.20	317.9 ± 15.9	304.3 ± 1.56	307.5 ± 10.8	289.7 ± 23.3	296.8 ± 11.6
BW final (g)	326.9 ± 19.9	361.2 ± 13.8	344.5 ± 44.2	392.7 ± 27.5	350.0 ± 8.59	357.7 ± 28.9	348.7 ± 40.8	331.9 ± 35.6
BW gain (total in 14 days)	19.7 ± 10.6	54.9 ± 6.86	44.9 ± 44.8	74.9 ± 17.2	45.7 ± 8.61	50.2 ± 19.5	59.0 ± 34.6	35.1 ± 25.7
BW gain (g/day)	1.41 ± 0.76	3.92 ± 0.49	3.21 ± 3.20	5.35 ± 1.23	3.26 ± 0.61	3.59 ± 1.40	4.22 ± 2.47	2.50 ± 1.83
liver weight (g)	11.3 ± 0.85 ^ab^	13.5 ± 0.38 ^ab^	10.2 ± 2.06 ^a^	14.8 ± 1.27 ^b^	12.7 ± 1.33 ^ab^	12.8 ± 1.25 ^ab^	13.1 ± 2.08 ^ab^	11.5 ± 2.54 ^ab^
kidney weight (g)	2.17 ± 0.12 ^ab^	2.23 ± 0.12 ^ab^	2.23 ± 0.06 ^ab^	2.63 ± 0.23 ^b^	2.20 ± 0.10 ^ab^	2.30 ± 0.26 ^ab^	2.37 ± 0.31 ^ab^	2.07 ± 0.12 ^a^
spleen weight (g)	0.67 ± 0.06 ^a^	0.77 ± 0.12 ^ab^	0.67 ± 0.06 ^a^	0.93 ± 0.06 ^ab^	0.83 ± 0.15 ^ab^	0.83 ± 0.06 ^b^	0.97 ± 0.12 ^ab^	0.70 ± 0.10 ^ab^
relative liver weight (%)	3.45 ± 0.09 ^ab^	3.75 ± 0.23	2.93 ± 0.23 ^a^	3.78 ± 0.14 ^b^	3.61 ± 0.29 ^b^	3.59 ± 0.13 ^ab^	3.76 ± 0.20 ^b^	3.45 ± 0.39 ^ab^
relative kidney weight (%)	0.67 ± 0.07	0.62 ± 0.05	0.66 ± 0.08	0.67 ± 0.01	0.63 ± 0.02	0.65 ± 0.10	0.68 ± 0.08	0.63 ± 0.04
relative spleen weight (%)	0.20 ± 0.01 ^ab^	0.21 ± 0.03 ^ab^	0.19 ± 0.02 ^a^	0.24 ± 0.03 ^ab^	0.24 ± 0.04 ^ab^	0.23 ± 0.04 ^b^	0.28 ± 0.02 ^b^	0.21 ± 0.03 ^ab^
Σ feed intake (g/14 days/ind.)	336.1 ± 6.41	405.8 ± 40.0	358.4 ± 43.1	448.7 ± 67.3	359.2 ± 2.71	382.2 ± 44.3	395.4 ± 69.1	363.7 ± 39.9

^a,b^ different small uppercase indices mean significant (*p* < 0.05) inter-group differences by ANOVA (bodyweight: BW).

**Table 2:** The hepatic total phospholipid fatty acid (PL FA) composition of the experimental rats (FD: FB_1_ + DON; FZ: FB_1_ + ZEA; DZ: DON + ZEA; FDZ: FB_1_ + DON + ZEA).

Group	Control	FB_1_	DON	ZEA	FD	FZ	DZ	FDZ
*PL FA profile of the LIVER*	Mean ± SD	Mean ± SD	Mean ± SD	Mean ± SD	Mean ± SD	Mean ± SD	Mean ± SD	Mean ± SD
C14:0	0.19 ± 0.05	0.22 ± 0.03	0.15 ± 0.07	0.22 ± 0.03	0.23 ± 0.01	0.22 ± 0.07	0.19 ± 0.01	0.18 ± 0.06
C15:0	0.17 ± 0.02	0.17 ± 0.01	0.13 ± 0.02	0.15 ± 0.03	0.19 ± 0.02	0.17 ± 0.06	0.18 ± 0.01	0.14 ± 0.03
C16:0	18.7 ± 0.88	20.3 ± 0.80	18.4 ± 1.08	20.1 ± 1.67	21.5 ± 0.47	19.4 ± 2.25	19.40 ± 1.41	19.3 ± 1.04
C16:1 n7	0.77 ± 0.35	1.28 ± 0.38	0.80 ± 0.54	1.75 ± 0.81	1.57 ± 0.22	1.54 ± 0.60	1.11 ± 0.31	1.00 ± 0.48
C17:0	0.42 ± 0.10	0.35 ± 0.02	0.38 ± 0.05	0.35 ± 0.07	0.29 ± 0.03	0.37 ± 0.11	0.39 ± 0.02	0.34 ± 0.03
C18:0	24.4 ± 1.22	23.1 ± 1.28	24.2 ± 1.74	22.9 ± 2.38	20.9 ± 0.40	22.2 ± 2.30	21.92 ± 1.42	22.8 ± 1.13
C18:1 n9c	2.52 ± 0.33	2.75 ± 0.39	2.60 ± 0.43	3.04 ± 0.70	2.62 ± 0.10	2.86 ± 0.35	2.52 ± 0.31	2.50 ± 0.10
C18:2 n6c	12.0 ± 1.90	12.3 ± 1.61	11.2 ± 1.34	11.6 ± 1.51	12.0 ± 0.31	10.2 ± 1.34	9.73 ± 0.98	10.7 ± 0.52
C18:3 n6	0.13 ± 0.08	0.15 ± 0.02	0.11 ± 0.03	0.26 ± 0.11	0.18 ± 0.02	0.20 ± 0.07	0.15 ± 0.02	0.15 ± 0.04
C18:3 n3	0.13 ± 0.07	0.07 ± 0.02	0.07 ± 0.03	0.06 ± 0.01	0.06 ± 0.01	0.07 ± 0.03	0.06 ± 0.01	0.09 ± 0.04
C20:0	0.06 ± 0.00 ^b^	0.03 ± 0.00 ^a^	0.03 ± 0.00 ^a^	0.04 ± 0.01 ^ab^	0.04 ± 0.01 ^a^	0.04 ± 0.01 ^ab^	0.04 ± 0.00 ^ab^	0.04 ± 0.01 ^ab^
C20:1 n9	0.11 ± 0.05	0.08 ± 0.02	0.08 ± 0.03	0.08 ± 0.01	0.08 ± 0.01	0.10 ± 0.02	0.11 ± 0.01	0.07 ± 0.03
C20:2 n7	0.38 ± 0.09	0.31 ± 0.04	0.29 ± 0.12	0.30 ± 0.09	0.37 ± 0.05	0.37 ± 0.09	0.42 ± 0.07	0.36 ± 0.08
C20.3 n6	0.72 ± 0.33	0.83 ± 0.09	0.69 ± 0.51	1.01 ± 0.18	0.96 ± 0.03	1.25 ± 0.25	1.02 ± 0.31	0.83 ± 0.41
C20:4 n6	30.9 ± 1.94	29.9 ± 1.02	32.4 ± 1.81	30.4 ± 1.87	30.1 ± 0.70	31.9 ± 1.88	32.97 ± 1.82	31.9 ± 0.78
C20:3 n3	0.08 ± 0.02	0.07 ± 0.03	0.09 ± 0.01	0.08 ± 0.01	0.09 ± 0.01	0.10 ± 0.01	0.10 ± 0.01	0.10 ± 0.00
C20:5 n3	0.18 ± 0.09	0.28 ± 0.07	0.18 ± 0.16	0.46 ± 0.15	0.37 ± 0.06	0.37 ± 0.13	0.28 ± 0.07	0.26 ± 0.15
C22:0	0.02 ± 0.01 ^b^	0.01 ± 0.00 ^ab^	0.01 ± 0.00 ^ab^	0.01 ± 0.00 ^a^	0.01 ± 0.01 ^ab^	0.01 ± 0.00 ^ab^	0.01 ± 0.00 ^ab^	0.01 ± 0.00 ^ab^
C24:0	0.02 ± 0.01	0.01 ± 0.01	0.01 ± 0.00	0.01 ± 0.00	0.01 ± 0.00	0.01 ± 0.00	0.01 ± 0.00	0.01 ± 0.00
C22:5 n3	1.00 ± 0.22 ^ab^	1.14 ± 0.11 ^ab^	0.95 ± 0.22 ^a^	1.11 ± 0.14 ^ab^	1.38 ± 0.14 ^b^	1.33 ± 0.05 ^ab^	1.29 ± 0.13 ^ab^	1.25 ± 0.12 ^ab^
C22:6 n3	7.23 ± 0.99	6.66 ± 1.66	7.20 ± 2.21	6.10 ± 1.00	7.07 ± 0.22	7.28 ± 0.99	8.11 ± 1.07	7.91 ± 0.40
saturated	43.9 ± 1.55	44.2 ± 1.04	43.3 ± 0.84	43.7 ± 0.84	43.2 ± 0.26	42.4 ± 0.38	42.14 ± 0.27	42.9 ± 0.12
MUFA	3.40 ± 0.07	4.11 ± 0.79	3.48 ± 0.65	4.86 ± 1.50	4.27 ± 0.32	4.50 ± 0.80	3.74 ± 0.63	3.58 ± 0.37
PUFA	52.7 ± 1.60	51.6 ± 1.12	53.2 ± 0.19	51.4 ± 1.47	52.5 ± 0.06	53.1 ± 1.12	54.12 ± 0.37	53.5 ± 0.41
n6	43.7 ± 2.75	43.1 ± 1.57	44.4 ± 2.63	43.3 ± 0.89	43.2 ± 0.42	43.5 ± 0.76	43.87 ± 1.00	43.6 ± 0.87
n3	8.62 ± 1.24	8.22 ± 1.64	8.48 ± 2.50	7.79 ± 0.95	8.97 ± 0.37	9.15 ± 0.83	9.83 ± 1.15	9.61 ± 0.41
n6/n3	5.16 ± 1.02	5.40 ± 1.18	5.70 ± 2.34	5.61 ± 0.71	4.83 ± 0.25	4.78 ± 0.45	4.51 ± 0.59	4.54 ± 0.28
UI	204.1 ± 3.52 ^ab^	199.1 ± 8.40 ^a^	207.7 ± 6.77 ^ab^	198.9 ± 8.33 ^a^	204.4 ± 0.55 ^ab^	210.2 ± 8.04 ^ab^	216.4 ± 3.62 ^b^	211.8 ± 0.36 ^ab^
ACL	18.57 ± 0.05	18.49 ± 0.10	18.60 ± 0.05	18.48 ± 0.12	18.50 ± 0.01	18.59 ± 0.13	18.65 ± 0.05	18.62 ± 0.03

^a,b^ different small uppercase indices mean significant (*p* < 0.05) inter-group differences by ANOVA (MUFA: monounsaturated FA; PUFA: polyunsaturated FA, ACL: average FA chain length).

**Table 3:** The renal total phospholipid fatty acid composition of the experimental rats (FD: FB_1_ + DON; FZ: FB_1_ + ZEA; DZ: DON + ZEA; FDZ: FB_1_ + DON + ZEA).

Group	Control	FB_1_	DON	ZEA	FD	FZ	DZ	FDZ
*PL FA profile of the KIDNEY*	Mean ± SD	Mean ± SD	Mean ± SD	Mean ± SD	Mean ± SD	Mean ± SD	Mean ± SD	Mean ± SD
C14:0	0.15 ± 0.01 ^a^	0.20 ± 0.02 ^ab^	0.19 ± 0.04 ^ab^	0.24 ± 0.03 ^ab^	0.26 ± 0.07 ^b^	0.21 ± 0.01 ^ab^	0.18 ± 0.03 ^ab^	0.19 ± 0.02 ^ab^
C15:0	0.20 ± 0.01	0.22 ± 0.02	0.22 ± 0.04	0.26 ± 0.04	0.30 ± 0.09	0.23 ± 0.02	0.22 ± 0.03	0.20 ± 0.03
C16:0	22.1 ± 0.45	21.6 ± 0.68	24.1 ± 2.58	26.2 ± 0.62	25.56 ± 4.32	22.2 ± 0.35	21.7 ± 1.92	21.9 ± 1.05
C16:1 n7	0.53 ± 0.14	0.61 ± 0.09	0.69 ± 0.13	0.69 ± 0.17	0.69 ± 0.29	0.80 ± 0.07	0.59 ± 0.15	0.66 ± 0.11
C17:0	0.30 ± 0.01	0.26 ± 0.10	0.28 ± 0.02	0.31 ± 0.09	0.30 ± 0.05	0.33 ± 0.08	0.32 ± 0.06	0.29 ± 0.07
C18:0	22.3 ± 0.12 ^ab^	22.9 ± 0.30 ^b^	21.0 ± 1.28 ^ab^	19.9 ± 0.66 ^a^	20.2 ± 2.28 ^ab^	21.9 ± 0.66 ^ab^	22.3 ± 0.72 ^ab^	21.9 ± 0.39 ^ab^
C18:1 n9c	5.23 ± 0.37	5.08 ± 0.35	5.63 ± 0.26	5.38 ± 0.64	5.10 ± 0.45	5.36 ± 0.31	4.95 ± 0.18	5.50 ± 0.39
C18:2 n6c	7.15 ± 0.50 ^ab^	8.02 ± 0.48 ^ab^	7.58 ± 1.09 ^ab^	8.76 ± 0.42 ^b^	8.45 ± 1.04 ^ab^	7.88 ± 0.41 ^ab^	6.94 ± 0.38 ^a^	7.36 ± 0.27 ^ab^
C18:3 n6	0.06 ± 0.01 ^a^	0.09 ± 0.02 ^ab^	0.07 ± 0.02 ^ab^	0.11 ± 0.01 ^b^	0.10 ± 0.03 ^ab^	0.08 ± 0.02 ^ab^	0.08 ± 0.00 ^ab^	0.08 ± 0.01 ^ab^
C18:3 n3	0.09 ± 0.01	0.06 ± 0.00	0.07 ± 0.01	0.06 ± 0.02	0.07 ± 0.01	0.06 ± 0.01	0.05 ± 0.01	0.07 ± 0.02
C20:0	0.10 ± 0.02	0.10 ± 0.02	0.09 ± 0.03	0.12 ± 0.01	0.10 ± 0.01	0.10 ± 0.02	0.10 ± 0.03	0.09 ± 0.01
C20:1 n9	0.08 ± 0.02 ^ab^	0.09 ± 0.02 ^ab^	0.07 ± 0.01 ^a^	0.12 ± 0.01 ^b^	0.07 ± 0.02 ^a^	0.10 ± 0.01 ^ab^	0.09 ± 0.02 ^ab^	0.09 ± 0.01 ^ab^
C20:2 n7	0.24 ± 0.04	0.26 ± 0.02	0.23 ± 0.06	0.30 ± 0.04	0.29 ± 0.06	0.23 ± 0.05	0.29 ± 0.07	0.29 ± 0.03
C20.3 n6	0.85 ± 0.21	0.98 ± 0.12	0.92 ± 0.29	1.12 ± 0.22	1.03 ± 0.13	1.14 ± 0.09	1.06 ± 0.08	0.95 ± 0.22
C20:4 n6	37.5 ± 0.68	36.5 ± 0.51	36.0 ± 3.33	33.5 ± 1.71	34.5 ± 3.46	36.2 ± 0.82	38.0 ± 1.88	37.3 ± 0.79
C20:3 n3	0.11 ± 0.01	0.10 ± 0.01	0.09 ± 0.01	0.10 ± 0.04	0.10 ± 0.00	0.09 ± 0.01	0.10 ± 0.02	0.08 ± 0.01
C20:5 n3	0.15 ± 0.02	0.19 ± 0.05	0.13 ± 0.03	0.17 ± 0.03	0.16 ± 0.01	0.19 ± 0.04	0.14 ± 0.01	0.17 ± 0.06
C22:0	0.05 ± 0.02	0.04 ± 0.00	0.03 ± 0.00	0.04 ± 0.00	0.03 ± 0.00	0.03 ± 0.01	0.03 ± 0.01	0.04 ± 0.01
C24:0	0.05 ± 0.00	0.06 ± 0.01	0.04 ± 0.01	0.06 ± 0.01	0.05 ± 0.02	0.04 ± 0.00	0.05 ± 0.02	0.04 ± 0.01
C22:5 n3	0.35 ± 0.03	0.31 ± 0.01	0.33 ± 0.06	0.31 ± 0.04	0.33 ± 0.05	0.36 ± 0.04	0.30 ± 0.01	0.37 ± 0.03
C22:6 n3	2.46 ± 0.08	2.30 ± 0.23	2.21 ± 0.32	2.24 ± 0.12	2.28 ± 0.13	2.49 ± 0.27	2.50 ± 0.10	2.42 ± 0.26
saturated	45.2 ± 0.42	45.4 ± 0.81	45.9 ± 1.44	47.2 ± 0.80	46.8 ± 2.45	45.0 ± 0.85	44.9 ± 1.25	44.7 ± 0.81
MUFA	5.84 ± 0.49	5.78 ± 0.45	6.40 ± 0.40	6.19 ± 0.80	5.86 ± 0.51	6.25 ± 0.38	5.63 ± 0.29	6.25 ± 0.31
PUFA	48.9 ± 0.08	48.8 ± 0.58	47.7 ± 1.84	46.7 ± 1.03	47.3 ± 2.60	48.7 ± 0.79	49.4 ± 1.52	49.1 ± 1.01
n6	45.5 ± 0.17	45.6 ± 0.76	44.6 ± 2.10	43.5 ± 1.19	44.1 ± 2.55	45.3 ± 1.00	46.1 ± 1.61	45.7 ± 0.82
n3	3.15 ± 0.10	2.96 ± 0.20	2.82 ± 0.40	2.88 ± 0.15	2.95 ± 0.18	3.20 ± 0.27	3.09 ± 0.08	3.10 ± 0.33
n6/n3	14.5 ± 0.52	15.5 ± 1.36	16.1 ± 2.90	15.2 ± 1.20	15.0 ± 1.13	14.3 ± 1.50	14.9 ± 0.90	14.8 ± 1.52
UI	191.1 ± 1.33	188.4 ± 1.21	185.1 ± 9.08	178.3 ± 4.23	181.5 ± 12.15	189.1 ± 1.88	193.1 ± 6.26	191.5 ± 4.53
ACL	18.43 ± 0.02	18.41 ± 0.01	18.34 ± 0.11	18.26 ± 0.04	18.29 ± 0.17	18.40 ± 0.01	18.45 ± 0.08	18.43 ± 0.05

^a,b^ different small uppercase indices mean significant (*p* < 0.05) inter-group differences by ANOVA (MUFA: monounsaturated FA; PUFA: polyunsaturated FA; ACL: average FA chain length).

**Table 4:** Antioxidant and lipid peroxidation parameters in the liver and kidney.

Group	Control	FB_1_	DON	ZEA	FD	FZ	DZ	FDZ
*Peroxidation traits, LIVER*	Mean ± SD	Mean ± SD	Mean ± SD	Mean ± SD	Mean ± SD	Mean ± SD	Mean ± SD	Mean ± SD
GSH (micromol/g protein)	2.11 ± 0.09 ^a^	2.60 ± 0.26 ^abc^	2.42 ± 0.52 ^ab^	3.34 ± 0.33 ^bc^	3.52 ± 0.13 ^c^	3.24 ± 0.35 ^bc^	3.38 ± 0.46 ^bc^	3.27 ± 0.43 ^bc^
GSHPx (IU/g protein)	0.99 ± 0.28 ^a^	1.53 ± 0.57 ^ab^	1.79 ± 0.55 ^ab^	1.70 ± 0.34 ^ab^	2.33 ± 0.29 ^b^	1.81 ± 0.22 ^ab^	1.82 ± 0.56 ^ab^	1.58 ± 0.39 ^ab^
MDA (micromol/g)	16.2 ± 0.34	17.6 ± 2.91	15.6 ± 3.99	15.6 ± 1.89	16.6 ± 1.52	15.0 ± 1.35	13.47 ± 0.71	16.0 ± 1.85
***Peroxidation traits, KIDNEY***								
GSH (micromol/g protein)	1.49 ± 0.24	1.76 ± 0.80	2.14 ± 0.50	1.98 ± 0.31	1.88 ± 0.25	1.99 ± 0.96	1.30 ± 0.32	1.92 ± 0.74
GSHPx (IU/g protein)	0.44 ± 0.11	0.59 ± 0.42	0.54 ± 0.15	0.42 ± 0.44	0.91 ± 0.57	0.93 ± 0.27	0.37 ± 0.23	0.73 ± 0.47
MDA (micromol/g)	4.13 ± 0.58 ^ab^	4.41 ± 0.35 ^ab^	5.78 ± 1.50 b	5.02 ± 0.97 ^ab^	4.31 ± 0.30 ^ab^	3.67 ± 0.14 ^a^	3.49 ± 0.49 ^a^	4.44 ± 0.26 ^ab^

^a,b,c^ different small uppercase indices mean significant (*p* < 0.05) inter-group differences by ANOVA.

**Table 5:** The chemical and fatty acid composition of the diet.

Chemical Composition	
Dry matter (%)	88.4
Crude protein (%)	19
Crude fat (%)	3.5
Crude fiber (%)	3.6
Crude ash (%)	6.5
N free extract (%)	55.9
Gross energy (MJ/kg)	16.4
Metab. energy (MJ/kg)	13.4
**Fatty Acid Composition**	**Weight %**
C14:0	0.01
C16:0	0.49
C16:1 n7	0.01
C18:0	0.08
C18:1 n9	0.65
C18:2 n6	1.9
C18:3 n3	0.25
C20:0	0.01
C20:1 n9	0.02
